# Meta-Analysis of the Prevalence of *Echinococcus* in Sheep in China From 1983 to 2020

**DOI:** 10.3389/fcimb.2021.711332

**Published:** 2021-07-26

**Authors:** Yang Gao, Wei Wang, Chuang Lyu, Xin-Yu Wei, Yu Chen, Quan Zhao, Zhi-Guang Ran, You-Qing Xia

**Affiliations:** ^1^ The Key Sericultural Laboratory of Agricultural Ministry, College of Biotechnology, Southwest University, Chongqing, China; ^2^ Research and Development Department, Chongqing Auleon Biological Co., Ltd., Chongqing, China; ^3^ College of Life Science, Changchun Sci-Tech University, Shuangyang, China; ^4^ College of Animal Science and Veterinary Medicine, Heilongjiang Bayi Agricultural University, Daqing, China; ^5^ Animal Health Center, Shandong New Hope Liuhe Group Co., Ltd., Qingdao, China; ^6^ Animal Health Center, Qingdao Jiazhi Biotechnology Co., Ltd., Qingdao, China

**Keywords:** *Echinococcus*, sheep, Echinococcosis, meta-analysis, China

## Abstract

Echinococcosis is a zoonosis caused by the larval stage of cestode species that belong to the genus *Echinococcus*. The infection of hydatid in sheep is very common in China, especially in the northwestern China. Here, we conducted the first systematic review and meta-analysis of echinococcosis in sheep in China. Six databases (PubMed, ScienceDirect, Baidu Library, CNKI, Wanfang, and VIP Chinese Journal Database) were used to retrieve the literatures on echinococcosis in sheep in China from 1983 to 2020, and 74 studies. The random effects model was used in the “meta” package of the R software and the PFT was chosen for rate conversion. The research data were analyzed through subgroup analysis and univariate meta-regression analysis to reveal the factors that lead to research heterogeneity. The combined prevalence of *Echinococcus* in the selected period was estimated to be 30.9% (192,094/826,406). In the analysis of sampling year, the lowest positive rate was 13.9% (10,296/177,318) after 2011. The highest prevalence of *Echinococcus* was 51.1% (278/531) in the southwestern China. The highest infection rate in sheep was 20.1% (58,344/597,815) in the liver. The analysis based on age showed that the infection rate of elderly sheep was significantly higher than that in younger animals (*P* < 0.05). We also evaluated the effects of different geographic and climatic factors on the prevalence of *Echinococcus* in sheep. The results showed that the prevalence of *Echinococcus* was higher in high altitude, cold, humid, and high rainfall areas. It is necessary to carry out long-term monitoring and control of echinococcosis, cut off the infection route, and reduce the risk of infection in the high risk areas.

## Introduction

Echinococcosis is a zoonosis caused by the larval stage of cestode species that belong to the genus *Echinococcus* (Villard et al., 2003). The disease is one of the 17 neglected tropical diseases (NTDs) recognized by the World Health Organization (Agudelo Higuita et al., 2016). Echinococcosis is a chronic infection disease in both animals and humans. This disease can take years before being noticed (Ohiolei et al., 2020b). Detection of hydatid infection is common during postmortem examination of animals and incidentally found in humans (Budke et al., 2013). *Echinococcus* is sometimes asymptomatic during its development stage except a cysts rupture for releasing antigenic material that causes reaction or active cysts located in certain anatomical regions (e.g., joints and eyes), and then exerts pressure on surrounding tissues, thus resulting in pain or discomfort (Budke, 2002; Kern et al., 2017).

Generally, the “Ingestion of contaminated food and water” and “direct contact/playing with dogs” are classically mentioned as the sources of human infection and are biologically plausible potential risk factors (Tamarozzi et al., 2019). Humans usually get infections from canines, the infection occurred in sheep directly reflects the endemicity degree and levels of human risk (Rashid et al., 2017). Intermediate hosts accidentally ingesting infective eggs that develop into a metacestode stage in different organs (such as liver, lung, and kidney), leading to echinococcosis. The predators can release infective eggs, which could lead to a contamination for the environment, thus threatening human health (Carmena et al., 2008; Assefa et al., 2015). Echinococcosis was identified as a limiting disease in livestock production, and the infected sheep would cause economic losses to some extent (Wahlers et al., 2012). Recent, a report showed that approximately 30 million livestock were affected by echinococcosis in China. An average of increase of echinococcosis was 7 million per year. Among all infected livestock, sheep occupied approximately 70%, and caused a total economic loss of approximately 1 billion Yuan (RMB) (Yu et al., 2008; Yang et al., 2015).

Notably, it was estimated that a total of one million disability-adjusted life years (DALYs) were caused by echinococcosis globally, out of which 0.40 million were in China (Qian et al., 2017). Additionally, echinococcosis could lead to a loss of US$ 1.92 billion globally per year, China was responsible for US$ 0.66 billion. The annual global livestock production losses associated with echinococcosis were also high, reaching US$ 2.19 billion, of which China occupied a great proportion (Budke et al., 2006; Qian et al., 2017). Sheep can provide meat, milk, and wool for human beings, thus becoming an important livestock in the world. With an increase of human population, the needs of sheep by-products have elevated worldwide (Zhu et al., 2018). China as a big sheep-raising country (Zhao and Zhang, 2019). Thus, it is necessary to estimate the prevalence of *Echinococcus* in sheep in China and identify potential risk factors for providing basic data for researchers. At present, there is no study on the potential risk factors of *Echinococcus* infection in sheep in China. Therefore, a systematic review and meta-analysis were conducted-required to determine the prevalence of *Echinococcus* in sheep in China and to assess potential risk factors (sampling site, region, infection organ, season, detection method, age, geographical location and climate factors, etc.).

## Materials and Methods

### Search Strategy and Selection Criteria

This study has been prepared according to the PRISMA guidelines for the design and analysis of selected qualified studies (**Table S1**) (Moher et al., 2009; Moher et al., 2015). The web of the six literature databases were employed to search for articles that related to the epidemiology of CE in sheep in China, including the China National Knowledge Infrastructure (CNKI), Baidu Library, PubMed, ScienceDirect, VIP Chinese Journal Database, and Wanfang Data. We searched all published papers with regard to CE in sheep from 1983 to December 20, 2020. We used MeSH terms “Echinococcosis”, “sheep” and “China”, as well as similar terms, such as “Echinococcoses”, “*Echinococcus* Infection”, “*Echinococcus* Infections”, “Infection, *Echinococcus*” “Cystic *Echinocccosis*”, “Cystic Echinocccoses”, “Echinocccoses, Cystic”, “*Echinocccosis*, Cystic”, “Hydatidosis”, “Hydatidoses”, “Cysts, Hydatid”, “Cyst, Hydatid”, “Hydatid Cysts”, “Hydatid Cyst”, “Hydatid Disease”, “Hydatid Diseases”, “*Echinococcus Granulosus* Infection”, “*Echinococcus Granulosus* Infections”, “*Granulosus* Infection, *Echinococcus*”, “*Granulosus* Infections, *Echinococcus*”, “Infection, *Echinococcus Granulosus*”, and “Infections, *Echinococcus Granulosus*”. Boolean operators “AND” and “OR” were used to connect MESH and entry terms. In PubMed, the search formula was ((((((((((((((((((((((((“Echinococcosis”[Mesh]) OR (Echinococcoses)) OR (*Echinococcus* Infection)) OR (*Echinococcus* Infections)) OR (Infection, *Echinococcus*)) OR (Cystic Echinocccosis)) OR (Cystic Echinocccoses)) OR (Echinocccoses, Cystic)) OR (Echinocccosis, Cystic)) OR (Hydatidosis)) OR (Hydatidoses)) OR (Cysts, Hydatid)) OR (Cyst, Hydatid)) OR (Hydatid Cysts)) OR (Hydatid Cyst)) OR (Hydatid Disease)) OR (Hydatid Diseases)) OR (*Echinococcus Granulosus* Infection)) OR (*Echinococcus Granulosus* Infections)) OR (*Granulosus* Infection, *Echinococcus*)) OR (*Granulosus* Infections, *Echinococcus*)) OR (Infection, *Echinococcus Granulosus*)) OR (Infections, *Echinococcus Granulosus*)) AND (((((((“Sheep”[Mesh]) OR (Ovis)) OR (Dall Sheep)) OR (Ovis dalli)) OR (Sheep, Dall)) OR (Sheep, Bighorn)) OR (Sheep, Domestic))) AND ((((((“China”[Mesh]) OR (People’s Republic of China)) OR (Mainland China)) OR (Manchuria)) OR (Sinkiang)) OR (Inner Mongolia)). In the Sciencedirect database, we searched for keywords sheep, Hydatid, Echinococcosis, Epidemiology, prevalence, China and the selected article type was research articles. In the four Chinese databases, “sheep” (in Chinese) and “Echinococcosis” (in Chinese) OR “sheep” (in Chinese) and “Hydatidosis” (in Chinese) were used as keywords for advanced search and were set to use synonym expansion or fuzzy search. We restricted the search to review and research articles and conference abstracts.

We adopted the following inclusion criteria: (1) the purpose of the study was to investigate the positive rate of *Echinococcus* in sheep; (2) the study provided the total number of sheep tested and the number of sheep that tested positive; (3) the study had a clear test method; (4) the research location was in China, and a precise sampling area was provided; (5) each sample was from one sheep and could not be mixed. Articles that did not meet these criteria were excluded. In addition, we did not contact the original authors to obtain more information, and unpublished data were not taken into account.

### Data Extraction

Three researchers individually used standardized data collection forms to extract the required data for the research. If the researchers held different views or expressed uncertainty about specific articles, these would be evaluated by a fourth researcher (Y.G., the main reviewer of the meta-analysis). The database was established using Microsoft Excel (version 16.39, Microsoft Corp., Redmond, WA, USA).

The following information was recorded: the first author, the total number of sheep samples examined and the number of positive samples, the year of publication, sampling time and location, the geographic data, the test method, age, gender, season, *Echinococcus* infection organs, *Echinococcus* species, and sample type. Statistical geographic factor data were obtained from the National Meteorological Information Center of China Meteorological Administration, including longitude range, latitude range, annual average rainfall, altitude, average yearly temperature, and average yearly humidity.

### Quality Assessment

The quality of the included studies was scored based on the GRADE criteria (Guyatt et al., 2008). The adopted criteria included random sampling, a precise sampling time, a clear detection method, a detailed sampling method, and an analysis containing four or more risk factors.

Each criterion was scored as 1 point. The total score was 5 points if a study met all mentioned criteria. Studies with 5 or 4 points were considered as high quality, studies with a score of 3 or 2 were considered as medium quality, and studies with a score of 1 or 0 were marked as low quality.

### Statistical Analysis

The R Studio software version 1.2.5019 (“R core team, R: A language and environment for statistical computing” R core team 2018) was used for data analysis (using the meta package). **Table S2** showed the code in R for this meta-analysis. Before conducting the meta-analysis, we tested four conversion methods to make the data closer to the Normal distribution, namely logarithmic conversion (PLN), logit transformation (PLOGIT), arcsine transformation (PAS), and double-arcsine transformation (PFT). After referring to the research of Wang et al., we chose PFT for rate conversion (Li et al., 2020; Wang et al., 2020). Due to the apparent heterogeneity of the included studies, we chose a random-effects model for meta-analysis. Forest plots were used for the overall assessment of meta-analysis. The funnel plot, trim and fill analysis, and Egger’s test were used to assess the publication bias of studies. A sensitivity analysis was conducted, and one study was deleted at a time to check whether any study would have a significant impact on the estimated results. Heterogeneity for studies was calculated by *Cochran-Q*, *I^2^* statistics, and *χ²* test. A *P-value* < 0.05 and an *I^2^* statistic with a cut-off of 50% were used to define a statistically significant degree of heterogeneity (Wei et al., 2021).

### Subgroup Analysis

In order to further study the potential sources of heterogeneity, the research data subjected to subgroup analysis and univariate meta-regression analysis were used to reveal the factors that led to a research heterogeneity. The boundary division in the subgroup was based on our statistical evaluation results to divide the cut-off value. The survey factors included the year of publication (after 2011 *vs*. before), geographic region (northeastern China *vs*. other regions), age (lamb *vs*. other age groups), gender (ewes *vs*. rams), detection method (ultrasonic test *vs*. other methods), season (autumn *vs*. other three seasons), infected organs (other *vs*. liver, both, lung), *Echinococcus* species (*E. granulosus vs*. *E. multilocularis*), and study quality (low quality *vs*. other levels of quality).

Besides, we assessed the impact of geographic risk factors on the study, including longitude (91-100° *vs*. other longitude ranges), latitude (30-35° *vs*. other longitude ranges), average yearly precipitation (401-1,000 mm *vs*. other precipitation groups), average yearly temperature (-5-0°C *vs*. other temperature ranges), average yearly humidity (61–68% *vs*. other humidity value groups), and altitude (30,001-100,000 dm *vs*. other altitude value groups).

## Results

### Search Results and Eligible Studies

**Figure 1:**
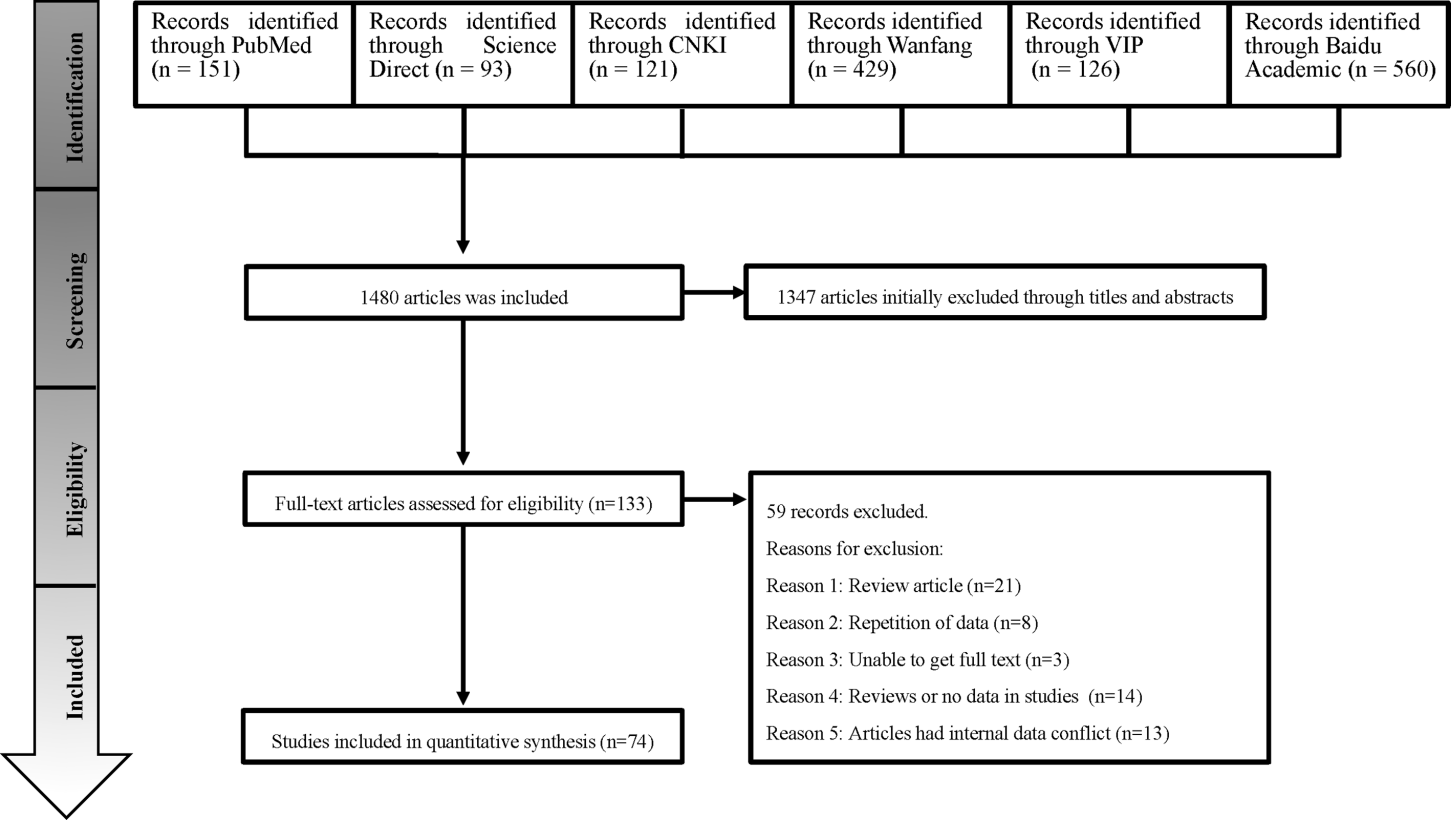
Flow diagram of the search strategies and selection of studies.

According to the inclusion and exclusion criteria, a total of 74 studies were used for meta-analysis by searching on six databases (**Figure 1**). 70 of them were from the Chinese database and 4 from the English database. Studies with 4 or 5 scores were considered as high-quality (23 studies), 2 or 3 scores as medium-quality (48 studies), and 0 or 1 score as low-quality research (3 studies; **Table S3**).

### Publication Bias and Sensitivity Analysis

**Figure 2:**
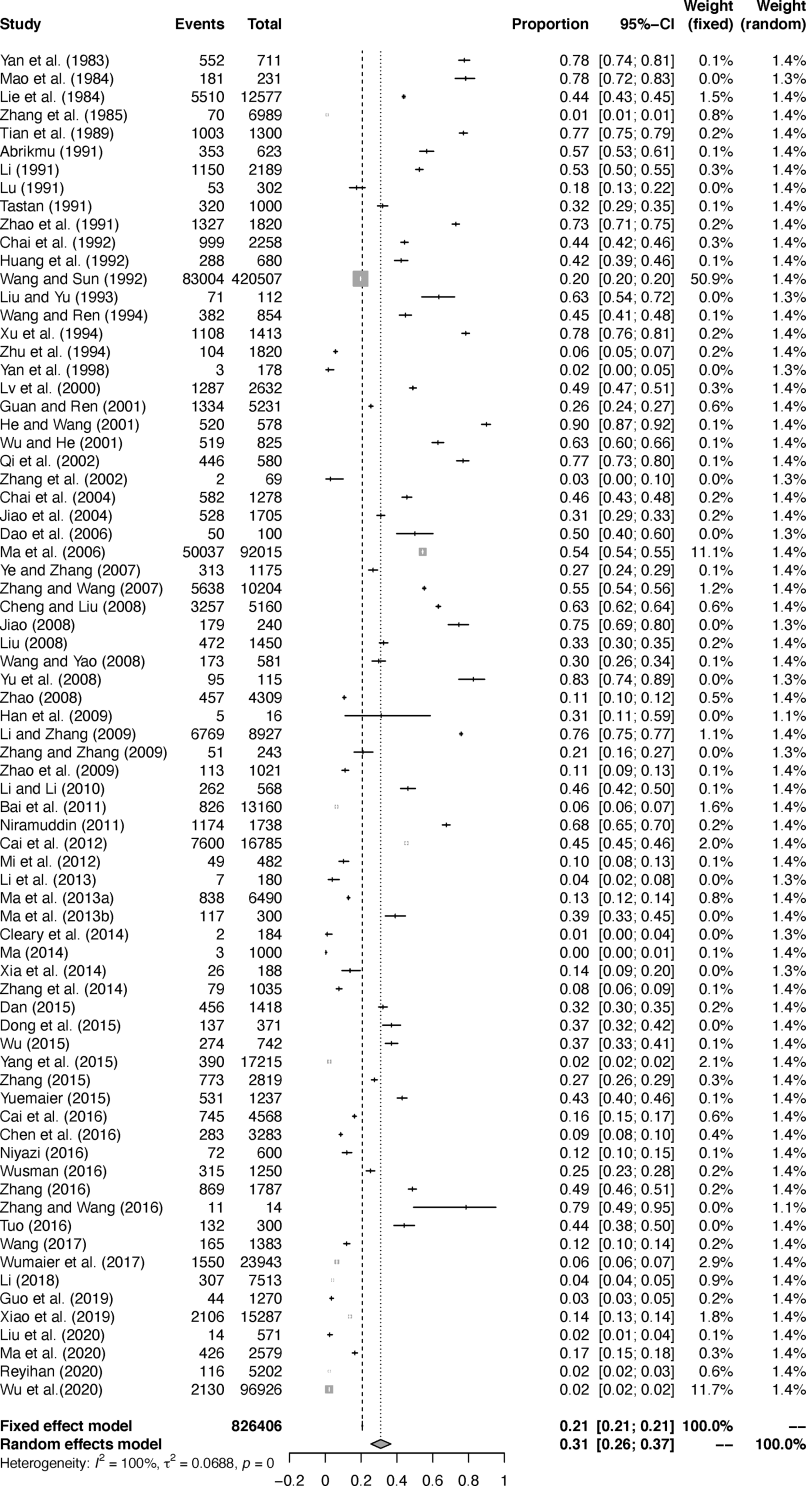
Forest plot of *Echinococcus* prevalence in sheep in China.

The extent of heterogeneity in the selected studies was measured and demonstrated by a forest plot (**Figure 2**). The funnel plot showed that the included studies might have publication biases (**Figure S1**). Meanwhile, the trim and fill analysis indicated a possible publication bias or a small-study effect in our study (**Figure S2**). However, *P* < 0.05 was found by an Egger’s test, manifesting that all the included studies may had publication bias (**Figure S3**; **Table S4**). According to the sensitivity tests, the combined prevalence was not significantly affected by any study that was omitted (**Figure S4**). These results validated that our analyses were reasonable and reliable.

### Pooling and Heterogeneity Analyses

**Figure 3:**
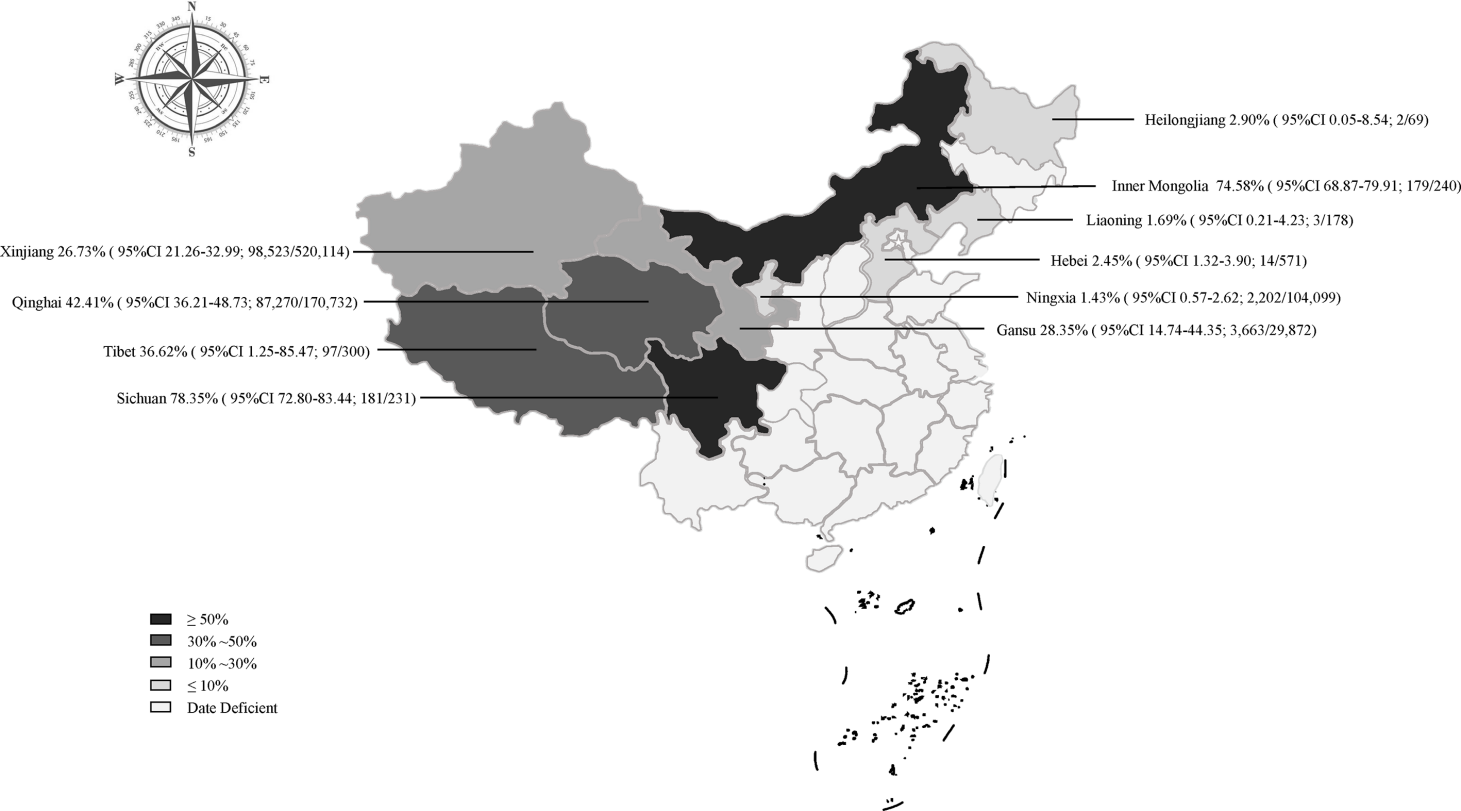
Map of *Echinococcus* prevalence in sheep in China.

In the selected studies, ten provinces were included (**Table 1**; **Figure 3**). In the subgroup analysis, we chose “PFT” for rate conversion data (**Table S5**), due to the high degree of heterogeneity in most subgroups, all estimated seroprevalence for each subgroup was calculated using random effect models (**Table 2**).

**Table 1:** Studies included in the analysis.

Study ID	Sampling time	Province	Detection method	Total samples	Positive samples	Quality score	Study Quality
Northeast China*							
Yan et al. (1998)	UN*	Liaoning	Anatomical touch detection	178	3	2	Medium
Zhang et al. (2002)	UN	Heilongjiang	Anatomical touch detection	69	2	3	Medium
Northern China*							
Jiao (2008)	UN	Inner Mongolia	UN	240	179	1	Low
Liu et al. (2020)	UN	Beijing	Serological testing	571	14	3	Medium
Northwest China*							
Abrikmu (2020)	UN	Xinjiang	Anatomical touch detection	623	353	2	Medium
Bai et al. (2011)	2007–2009	Gansu	Anatomical touch detection	13,160	826	3	Medium
Cai et al. (2012)	1990–2010	Qinghai	Anatomical touch detection	16,785	7,600	3	Medium
Cai et al. (2016)	UN	Qinghai	Anatomical touch detection	4,568	745	3	Medium
Chai et al. (1992)	1990–1992	Xinjiang	Anatomical touch detection	2,258	999	4	High
Chai et al. (2004)	2000–2002	Xinjiang	Anatomical touch detection	1,278	582	3	Medium
Chen et al. (2016)	2011–2012	Xinjiang	Anatomical touch detection	3,283	283	3	Medium
Cheng and Liu (2008)	1997–2001	Qinghai	Anatomical touch detection	5,160	3,257	3	Medium
Cleary et al. (2014)	2011	Ningxia	Anatomical touch detection	184	2	4	High
Dan (2015)	2004 / 2012–2014	Qinghai	Anatomical touch detection	1,418	456	1	Low
Dao et al. (2006)	UN	Gansu	Anatomical touch detection	100	50	2	Medium
Dong et al. (2015)	2014.07	Xinjiang	Ultrasonic testing	371	137	3	Medium
Guan and Ren (2001)	UN	Qinghai	Anatomical touch detection	5,231	1,334	2	Medium
Guo et al. (2019)	2013.05–2016.05	Xinjiang	Anatomical touch detection	1,270	44	3	Medium
Han et al. (2019)	2007.08–09	Qinghai	Anatomical touch detection	16	5	3	Medium
He and Wang (2001)	2000.06–09	Qinghai	Anatomical touch detection	578	520	3	Medium
Huang et al. (1992)	1991.09	Qinghai	Anatomical touch detection	680	288	3	Medium
Jiao et al. (2004)	2000–2003	Xinjiang	Anatomical touch detection	1,705	528	2	Medium
Li (2009)	2003.02–2008.10	Qinghai	Anatomical touch detection	2,189	1,150	2	Medium
Li (2018)	2016.01–12	Gansu	Anatomical touch detection	7,513	307	5	High
Li and Li (2010)	2009.02–10	Qinghai	Anatomical touch detection	568	262	2	Medium
Li and Zhang (2009)	1984 / 1997 / 2006	Qinghai	Anatomical touch detection	8,927	6,769	3	Medium
Li et al. (2013)	2013	Xinjiang	Ultrasonic testing	180	7	4	High
Lie et al. (1984)	1982.11–12	Qinghai	Anatomical touch detection	12,577	5,510	3	Medium
Liu (2008)	2007.01–08	Qinghai	Anatomical touch detection	1,450	472	4	High
Lu (2015)	2009.03–2009.05	Qinghai	Anatomical touch detection	302	53	2	Medium
Lv et al. (2000)	1999.09–1999.10	Qinghai	Anatomical touch detection	2,632	1,287	2	Medium
Ma (2014)	2012	Qinghai	Anatomical touch detection	1,000	3	4	High
Ma et al. (2006)	1997–2001	Qinghai	Anatomical touch detection	92,015	50,037	3	Medium
Ma et al. (2013a)	2012.10–12	Xinjiang	Anatomical touch detection	6,490	838	3	Medium
Ma et al. (2013b)	2012	Xinjiang	Anatomical touch detection	300	117	3	Medium
Ma et al. (2020)	UN	Xinjiang	Anatomical touch detection	2,579	426	3	Medium
Mi et al. (2012)	2011.09–10	Qinghai	Anatomical touch detection	482	49	4	High
Niramuddin (2011)	2010	Xinjiang	Anatomical touch detection	1,738	1,174	4	High
Niyazi (2016)	2015	Xinjiang	Anatomical touch detection	600	72	4	High
Qi et al. (2002)	1991–1993	Gansu	Serological testing	580	446	3	Medium
Reyihan (2020)	2019	Xinjiang	Anatomical touch detection	5,202	116	3	Medium
Tastan (2011)	2009.07–09	Xinjiang	Anatomical touch detection	1,000	320	5	High
Tian et al. (1989)	UN	Gansu	Anatomical touch detection	1,300	1,003	2	Medium
Tuo (2016)	2016.03–05	Qinghai	Anatomical touch detection	300	132	5	High
Wang (2017)	2016	Xinjiang	Anatomical touch detection	1,383	165	4	High
			& Serological testing				
Wang and Ren (1994)	UN	Gansu	UN	854	382	3	Medium
Wang and Sun (1992)	1980–1987	Xinjiang	Anatomical touch detection	420,507	83,004	2	Medium
Wang and Yao (2008)	2005–2006	Qinghai	Anatomical touch detection	581	173	4	High
Wu (2015)	2014.07	Xinjiang	Ultrasonic testing	742	274	3	Medium
Wu and He (2001)	1989–1991	Qinghai	Anatomical touch detection	825	519	4	High
Wu et al. (2020)	2011–2018	Ningxia	Anatomical touch detection	96,926	2,130	3	Medium
Wumaier et al. (2017)	2012.08–2013.09	Xinjiang	Anatomical touch detection	23,943	1,550	3	Medium
Wusman (2016)	UN	Xinjiang	Anatomical touch detection	1,250	315	2	Medium
Xiao et al. (2019)	2014–2017	Xinjiang	Anatomical touch detection	15,287	2,106	5	High
			& Serological testing				
Xu et al. (1994)	1992–1993	Xinjiang	Anatomical touch detection	1,413	1,108	4	High
Yan et al. (1983)	UN	Qinghai	UN	711	552	1	Low
Yang et al. (2015)	2007–2013	Xinjiang	Anatomical touch detection	17,215	390	3	Medium
Ye and Zhang (2007)	2006.09–2006.10	Qinghai	Anatomical touch detection	1,175	313	4	High
Yuemaier (2015)	UN	Xinjiang	Anatomical touch detection	1,237	531	4	High
Yu et al. (2008)	2005.07	Qinghai	Anatomical touch detection	115	95	2	Medium
Zhang (2015)	2011–2015	Xinjiang	Anatomical touch detection	2,819	773	3	Medium
Zhang (2016)	2016	Xinjiang	Serological testing	1,787	869	5	High
Zhang et al. (1985)	UN	Ningxia	Anatomical touch detection	6,989	70	4	High
Zhang and Wang (2007)	1990–2005	Qinghai	Anatomical touch detection	10,204	5,638	5	High
Zhang and Wang (2016)	2015.09–12	Xinjiang	Anatomical touch detection	14	11	4	High
Zhang and Zhang (2009)	2007.08–2008.05	Qinghai	Anatomical touch detection	243	51	3	Medium
Zhang et al. (2014)	2009.08/2010.09	Gansu	Anatomical touch detection	1,035	79	3	Medium
Zhao (2008)	2005–2007	Gansu	Anatomical touch detection	4,309	457	4	High
Zhao et al. (1991)	1990.09–10	Xinjiang	Anatomical touch detection	1,820	1,327	3	Medium
Zhao et al. (2009)	2005.07	Gansu	Anatomical touch detection	1,021	113	3	Medium
Zhu et al. (1994)	1990.02–1992.02	Xinjiang	Anatomical touch detection	1,820	104	2	Medium
Southwest China*							
Liu and Yu (1994)	UN	Xinjiang	UN	112	71	2	Medium
Mao et al. (1984)	1982.11	Sichuan	Anatomical touch detection	231	181	3	Medium
			& Serological testing				
Xia et al. (2014)	UN	Tibet	Anatomical touch detection	188	26	3	Medium

Northeast China*: Heilongjiang, Jilin, Liaoning.

North China*: Beijing, Tianjin, Hebei, Shanxi, Inner Mongolia.

Northwest China*: Shaanxi, Gansu, Qinghai, Ningxia, Xinjiang.

Southwest China*: Chongqing, Sichuan, Guizhou, Yunnan, Tibet.

UN*: unclear.

**Table 2:** The combined prevalence of *Echinococcus* infection in sheep in China.

		No. studies	No. examined	No. positive	% (95% CI*)	Heterogeneity			Univariate meta-regression	
						*χ* ^2^	*P*-value	*I^2^*(%)	*P*-value	Coefficient (95% CI)
**Season**									0.018	0.209 (0.036 to 0.382)
	Autumn	12	104,273	54,310	44.70% (30.93–58.88)	5,543.24	0.00	99.8		
	Summer	10	8,468	1,778	31.07% (14.95–49.94)	2,235.77	0.00	99.6		
	Winter	4	26,340	1,939	19.48% (10.59–30.26)	265.91	< 0.01	98.9		
	Spring	4	886	186	15.42% (3.65–33.06)	113.17	< 0.01	97.3		
Age*									0.016	-0.309 (-0.560 to -0.057)
	Old sheep	10	2,887	1,203	35.89% (21.38–51.82)	632.93	< 0.01	98.6		
	Adult sheep	27	122,919	55,883	24.67% (14.00–37.20)	27,211.82	0.00	99.9		
	Lamb	8	14,362	411	5.55% (1.56–11.70)	931.52	< 0.01	99.2		
**Sampling**									<0.0001	-0.325 (-0.447 to -0.203)
**year**	2000 o rbefore	17	541,354	148,835	55.91% (42.96–68.46)	69106.07	0.00	100		
	2001 to 2010	22	58,989	16,077	33.23% (21.32–46.34)	20,590.54	0.00	99.9		
	2011 or late	23	177,318	10,269	13.86% (9.94–18.3)	9,917.86	0.00	99.8		
**Method**									0.644	-0.072 (-0.376 to 0.232)
	Serological testing	5	9,855	2,413	28.44% (9.33–52.9)	1,781.97	0.00	99.8		
	Anatomical touch detection	66	814,195	188,501	29.77% (24.09–35.79)	158,630.05	0.00	100		
	Ultrasonic testing	3	1,293	418	23.40% (7.11–45.38)	124.98	< 0.01	98.4		
Region*									0.022	-0.438 (-0.813 to -0.063)
	Southwestern	3	531	278	51.09% (11.51–89.86)	212.49	< 0.01	99.1		
	Northern	2	811	193	31.85% (0.00–99.00)	525.95	< 0.01	99.8		
	Northwestern	67	824,817	191,658	31.23% (25.53–37.23)	160,941.08	0.00	100		
	Northeastern	2	247	5	1.89% (0.42–4.15)	0.49	0.49	0		
**Gender**									0.885	0.024 (-0.298 to 0.346)
	Ram	3	1,855	1,048	51.55% (32.72–70.16)	24.74	< 0.01	91.9		
	Ewe	6	4,156	2,155	48.86% (30.51–67.36)	635.87	< 0.01	99.2		
**Infected organs**										
	Liver	34	597,815	58,344	20.10% (15.19–25.51)	40,471.45	0.00	99.9	0.004	-0.246 (-0.415to -0.076)
	Both*	25	154,439	58,206	18.87% (8.92–31.44)	53,576.89	0.00	100		
	lung	29	595,469	58,206	8.23% (4.42–13.05)	51,471.17	0.00	99.9		
	other	7	120,420	2,505	2.50% (0.00–10.62)	8,995.12	0.00	99.9		
**Echinococcus species**									0.0817	-0.427 (-0.907 to 0.054)
	Echinococcus granulosus	9	15,415	5,306	32.05% (11.49–57.10)	7,626.00	0.00	99.9		
	Echinococcus multilocularis	3	2,175	65	2.99% (0.19–8.74)	68.09	< 0.01	97.1		
**Sample type**									0.990	0.001 (-0.205 to 0.207)
	Serum	7	14,296	2,666	30.44% 13.85–50.19)	2,934.59	0.00	99.8		
	Organs	71	812,110	189,468	30.52% (24.97–36.37)	158,932.62	0.00	100		
**Sampling location**									0.016	0.204 ( 0.038 to 0.370)
	Pasture	18	16,547	4,253	39.82% (21.45–59.78)	9,520.34	0.00	99.8		
	Slaughterhouse	48	699,590	138,966	29.74% (22.60–37.42)	141,267.97	0.00	100		
**Quality level**									0.034	0.330 (0.026 to 0.633)
	Low	3	2,369	1187	62.05% (27.76–90.63)	494.6	< 0.01	99.6		
	Medium	48	762,128	175,450	31.23% (24.48–38.41)	140,929.80	0.00	100		
	High	23	61,909	15,497	26.63% (16.46–38.23)	196,36.73	0.00	99.9		
	Total	74	826,406	192,094	30.94% (25.51–36.64)					

CI*: Confidence interval;

NA*: Not applicable;

Age*: Lamb (< 1 year); Adult sheep (1–6 years old); Old sheep (> 6 years old).

Both*: Mixed liver and lung infection.

The prevalence was significantly different in different regions. The southwestern China had the highest prevalence (50.1%), and northeastern China had the lowest prevalence (1.9%). The pooled prevalence of *Echinococcus* in sheep ranged from 1.4% to 78.4% in different provinces (**Figure 3**). In the provinces, Sichuan kept the highest prevalence of 78.4%, and Ningxia was the lowest (1.4%; **Figure 3**).

Our findings showed that the prevalence of *Echinococcus* was higher in studies with sampling site from pasture (39.8%) than slaughterhouse (29.7%). Among these studies, the highest prevalence of *Echinococcus* based on sampling time was 55.9% in 2000 or before, and the lowest prevalence was 13.9% in 2011 or later. The highest detectable rate of *Echinococcus* was 20.1% in samples from liver, and the lowest was 2.5% in other. The prevalence of *Echinococcus* in elderly sheep was the highest (35.9%), and the lowest in young sheep (5.6%; **Table 2**).

Detailed geographical and climatic factors were further analyzed. The results showed that the prevalence of *Echinococcus* at altitude range (3,000-100,000m; 50.7%), rainfall range (401-1,000mm; 43.5%); latitude range (30-35°; 46.9%); longitude range (91-100°; 42.7%); minimum annual mean temperature range (< -5°C; 68.8%); average annual maximum temperature range (0-10°C; 53.0%); temperature range (-5-0°C; 58.7%), and humidity range (61-68; 38.5%) were higher than those in other ranges (**Table S6**).

## Discussion

For the first time, we conducted a systematic review and meta-analysis of the infection of *Echinococcus* sheep in China. The statistical results show that season, age, region, infected organization, sampling location, sampling year, article quality and geographic factors (precipitation, temperature and altitude) may be risk factors for sheep infection.

According to the statistics, the combined prevalence of *Echinococcus* in sheep in China was 30.9% (**Figure 2**), which was higher than 12.1% in Africa (Ohiolei et al., 2020a) and 8.8% in Ethiopia (Asmare et al., 2016), in published meta-analysis. Thus, it requires us to pay enough attention and take certain measures to prevent the disease. In regard to the sampling year subgroup, the prevalence of *Echinococcus* in sheep had a significant downward trend, and the combined prevalence after 2011 was the lowest (13.9%, *P* < 0.05; Table 2). In 2012, in accordance with the mission objectives of the “Medium and Long-term Animal Disease Prevention and Control Plan (2012-2020)” issued by China, each region formulated a series of comprehensive prevention and control measures based on the actual situation of echinococcosis in local animals (Zhang, 2016). The measures include immunization of newborn lambs in key areas, deworming of dogs in pastoral areas, and harmless treatment of diseased animal organs in slaughterhouses, etc. The implementation of these measures has played a key role in reducing sheep infections.

Among the included articles, most of the epidemiological investigations were concentrated in the northwestern region (91%; Table 2). The northwestern region was the main breeding area for sheep in China and was also a high-epidemic area for echinococcosis (Han et al., 2019), among which Qinghai province has the highest prevalence rate (**Figure 3**). From the perspective of geographic environment, the altitude subgroup analysis showed a relatively high prevalence of *Echinococcus* among sheep in high altitude areas, such as Qinghai province (*P* < 0.05; **Table S6**). Qinghai province has a plateau continental climate with sufficient sunshine and little precipitation, and the strong wind can provide condition for the spread of insect eggs on the pasture. In addition, the land is vast and rich in animal resources. Many domestic animals and rodents can serve as natural intermediate hosts for echinococcosis, thus providing favorable condition for the spread of the disease (Wen et al., 2019). From the perspective of feeding habits, the local herders are used to raising herding dogs and herding sheep, and often feed the dogs with the organs of dead sheep, causing a large number of infections in dogs (Yang et al., 2015). After an infection, the *Echinococcus* eggs in the dog feces contaminate the pasture and the sheep are infected. This makes a completion of the life cycle of *Echinococcus* in livestock. In addition, several surveys showed that the infection rate of *Echinococcus* in dogs, foxes, and rodents in Qinghai province was relatively high (Cai et al., 2016), indicating that the environment in this area was highly contaminated by *Echinococcus* eggs. It also indicated that there was a food chain relationship among the infected animals, which forms the cycle chain of life history (Wen et al., 2019). The production tradition and geographical environment of the local herders have caused a high incidence of *Echinococcus*, bringing a great difficulty to the prevention and control work. It is recommended that the health department in this area strengthen the herders’ awareness of livestock breeding and disease prevention, and regularly feed dogs with anthelmintics and not feed the internal organs of animals in order to cut off the path of infection.

In the seasonal subgroup, the prevalence of *Echinococcus* in sheep was higher in summer and autumn, but the size of data in the subgroup was relatively small. Therefore, we combined geographical factors (temperature, humidity and precipitation subgroups) to specifically analyze the suitable living condition for *Echinococcus* eggs and the impact on the prevalence of *Echinococcus* in sheep. According to the results, the prevalence of sheep was higher in the range of 91-100° longitude and 30-35° latitude and in cold, wet, and rainy areas (**Table S6**). *Echinococcus* eggs were extremely resistant to cold and can maintain vigor in ice and snow. A Swiss study showed that a low temperature may be positively correlated with the infection rate of the intermediate hosts of *Echinococcus* (Burlet et al., 2011). Consistent with our research results. In winter, herders have the habit of using melted ice and snow as drinking water, making ice and snow contaminated by insect eggs the main sources of echinococcosis in humans and animals (Zhao, 2008). The eggs of *Echinococcus* were very sensitive to dryness and high temperature. The infection rate of multilocular echinococcosis in Slovakia was obviously positively correlated with the amount of precipitation. in addition, the seasonal changes can cause stress responses to the animal body. It will affect the prevalence of hydatid disease. For example, in Zurich, the infection rate of *E. multilocularis* in young foxes was highest in winter, 56.75%, and lowest in spring, 13.20% (Li, 2018). We speculate that the geographical and climatic factors may be the risk factors for hydatid infection in sheep, and it is recommended that herders in high-cold and humid areas should pay more attention to the safety of water sources.

Some studies showed that the infection of *Echinococcus* might be related to the age and immunity of livestock (Yang et al., 2015). Therefore, we conducted a subgroup analysis to investigate whether there was a correlation between age and sex of host and *Echinococcus* infection. The subgroup data showed that the highest prevalence rate of *Echinococcus* in elderly sheep was 35.89%, which was much higher than 5.55% of young sheep, and the infection rate was positively correlated with an increase of age (*P* < 0.05; Table 2). Similar results have been found in other studies, showing the prevalence of *Echinococcus* in animals over 5 years old was higher (Cabrera et al., 2003; Azlaf et al., 2006). It is generally believed that with the increase of age, the chance of exposure to pathogens is increased, which makes the infection rate of elderly sheep higher than that of lambs and adult sheep. In the gender subgroup, the prevalence of *Echinococcus* in rams was slightly higher than that of ewes, but no significant difference was observed (*P* = 0.88; Table 2). In the subgroup of infected organs in sheep, it was shown that the *Echinococcus* infection was involved in different organs, with the liver being the most susceptible organ, the highest infection rate was 21.10%. A meta-analysis in Iran showed the same results, with the highest infection rate in the liver of 55% (Mahmoudi et al., 2019). A systematic review of the literature of human cystic *Echinococcus* (CE) indicated that *E. granulosus sensu stricto* metacestodes preferentially developed in the liver (73.4%), and secondly in the lungs (19.6%), with the remainder organs including the brain, spleen, kidney, and heart (Kern et al., 2017). This result can be explained by the fact that the liver and lung were the most important body filters and were the first sites to encounter the migrating parasite larvae, and a few parasites can escape from them and gain access to other organs (Ahmadi and Badi, 2011). From the perspective of the types of hydatid, the infection rate of *E. granulosus* was higher than that of *E. multilocularis*, but only a few articles recorded the types of *Echinococcus.* This may not reflect the true situation.

At present, the investigation of sheep *Echinococcus* is still mainly based on the on-site inspection of the slaughterhouse recommended by OIE. Most of the samples (90%) tested in the study were derived from organs, and a small part (10%) of the samples were serum (**Table 2**). Among them, the anatomical touch method has the highest detection rate, and a small number of them used serology and ultrasound methods. Visceral hydatid cyst inspection can only be performed at the time of livestock slaughter, which has a great limitation. In contrast, the application of serological antibody detection methods is superior to traditional detection methods in sensitivity, specificity, and practicability. Commercial ELISA kits were widely used in a large-scale epidemiological investigation (Siles Lucas et al., 2017), but studies have also shown false negatives and false positives, in addition to low repetition rates (Paul and Stefaniak, 2001; Auer et al., 2009), whereas western blot results showed a better sensitivity (Liance et al., 2000). Imaging techniques are essential for diagnosis, with benefits of relatively inexpensive cost. The portable ultrasound was widely used to diagnose CE liver lesions; X-ray was used for lung cysts (Solomon et al., 2018; Tamarozzi et al., 2018). Ultrasound diagnosis of liver echinococcosis has been employed in China since 1950s. Ultrasound examination is a non-invasive, painless, reproducible, and highly accurate examination method. The diagnostic accuracy rate of ultrasound is as high as 97.2%, and it can be used for an early diagnosis and differential diagnosis of echinococcosis (Zhao, 2008). Therefore, the combination of serological, clinical, and imaging methods is the most suitable diagnostic approach for echinococcosis.

The 74 investigated studies overall were of high quality, among which 23 high-quality studies accounted for 31%, and 48 medium-quality studies accounted for 65% (**Table S3**). The main reason for the loss of scores in low and medium-quality research was that the sampling method was not described in detail or a random sampling. Thus, it is recommended that researchers should record and analyze the actual situation in detail when conducting epidemiological investigations and in-depth excavation, and analysis of the specific causes of sheep infection, in order to provide accurate data for the study of echinococcosis.

This study conducted a comprehensive and detailed analysis of the risk factors for the epidemiology of *Echinococcus* in sheep in China. However, some limitations were also present in this study. First, although we have established a comprehensive search method, omissions may still exist. Secondly, lack of data in some regions, heterogeneity among studies, and insufficient research on certain subgroups (such as the species infected with *Echinococcus* and the gender subgroup of sheep) may affect the results of the analysis. Despite these limitations, this report has reflected an actual prevalence of echinococcosis in sheep in China.

## Conclusions

In the past three decades, the prevalence of *Echinococcus* in sheep in China has declined. However, the infection of *Echinococcus* in sheep in China is still severe, according to the published data. We comprehensively analyzed various risk factors affecting the prevalence of hydatid cysts and found that the prevalence rate was higher in high-altitude, cold, humid and rainy areas. More attention should be paid to the prevention and control of echinococcosis in the northwestern region that meets the conditions for oocyst survival and is dominated by animal husbandry. Due to a serious effect of echinococcosis on the livestock and poultry breeding industry, and a threat for human health, it is necessary to implement long-term monitoring and control measures for echinococcosis, cut off the path of infection to reduce the risk of human infection.

## Data Availability Statement

The original contributions presented in the study are included in the article/**Supplementary Material**. Further inquiries can be directed to the corresponding authors.

## Author Contributions

Y-QX, Z-GR, and QZ were responsible for the idea and concept of the paper. WW, X-YW, and YC built the database. WW and YG analyzed the data. YG wrote the manuscript. CL critically reviewed and revised the manuscript. All authors contributed to the article and approved the submitted version.

## Funding

This work was supported by the Key Scientific and Technological Achievements Transformation Project of Jilin Province (20170307016NY). The authors declare that this study received funding from Key Scientific and Technological Achievements Transformation Project of Jilin Province (20170307016NY). The funder had the following involvement in the study: Quan Zhao. Quan Zhao was responsible for the idea and concept of the paper.

## Conflict of Interest

YG and Z-GR were employed by Chongqing Auleon Biological Co., Ltd. CL was employed by Shandong New Hope Liuhe Group Co., Ltd., and Qingdao Jiazhi Biotechnology Co., Ltd.

The remaining authors declare that the research was conducted in the absence of any commercial or financial relationships that could be construed as a potential conflict of interest.

## Publisher’s Note

All claims expressed in this article are solely those of the authors and do not necessarily represent those of their affiliated organizations, or those of the publisher, the editors and the reviewers. Any product that may be evaluated in this article, or claim that may be made by its manufacturer, is not guaranteed or endorsed by the publisher.
