# Supplementary material for: Meta-Analysis of the Prevalence of Echinococcus in Sheep in China From 1983 to 2020
Source: Front Cell Infect Microbiol. 2021 Jul 26;11:711332. doi: 10.3389/fcimb.2021.711332 (PMC8350519; doi:10.3389/fcimb.2021.711332)
Supplement: Supplementary file 1 [file DataSheet_1.pdf]

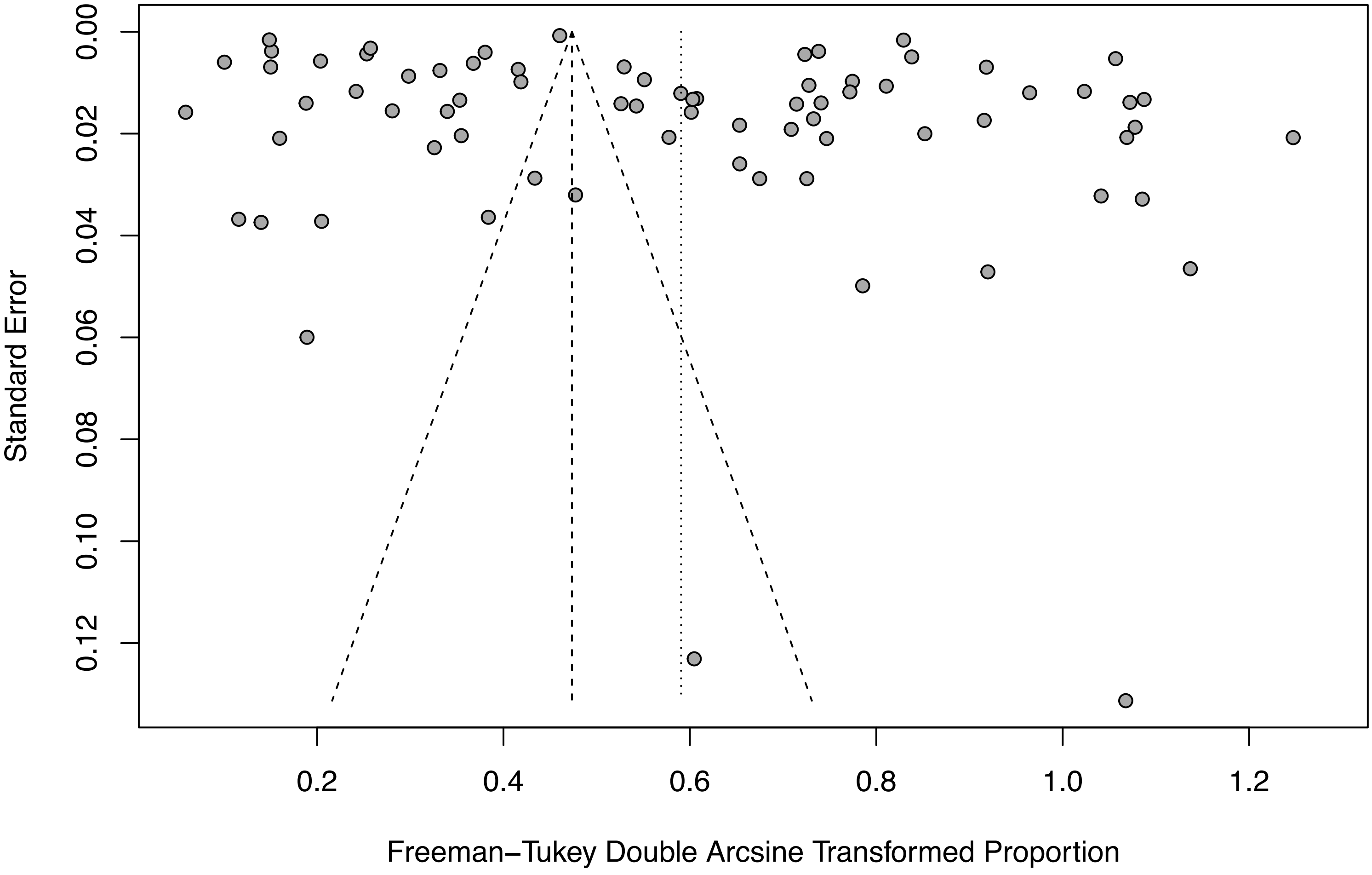

Figure S1 Funnel plot with pseudo 95% confidence interval limits for the examination of publication bias
