# Supplementary figures and images for: Meta-Analysis of the Prevalence of Echinococcus in Sheep in China From 1983 to 2020
Source: Front Cell Infect Microbiol. 2021 Jul 26;11:711332. doi: 10.3389/fcimb.2021.711332 (PMC8350519; doi:10.3389/fcimb.2021.711332)

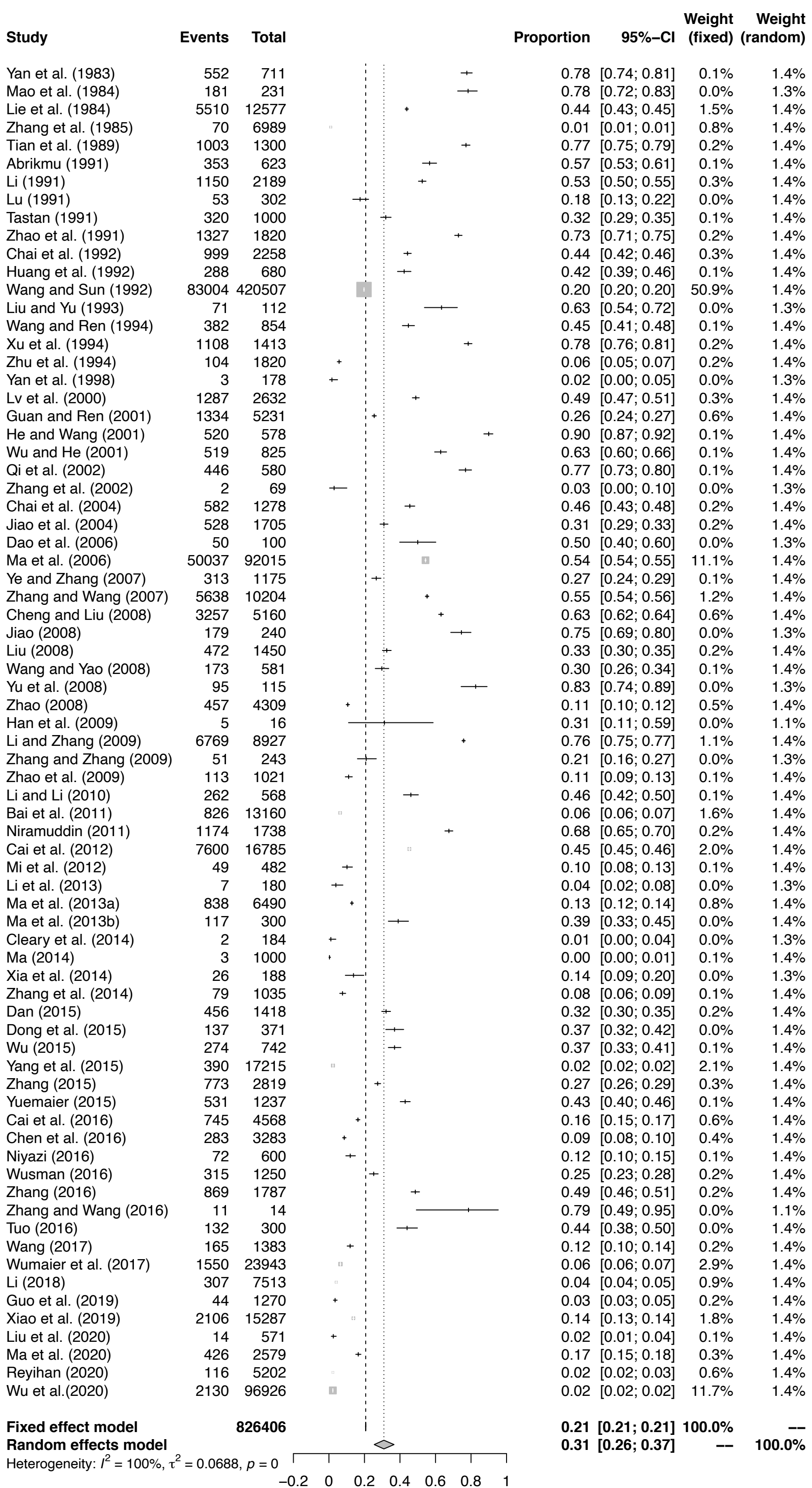

Supplement: Supplementary file 2 [file DataSheet_2.pdf]

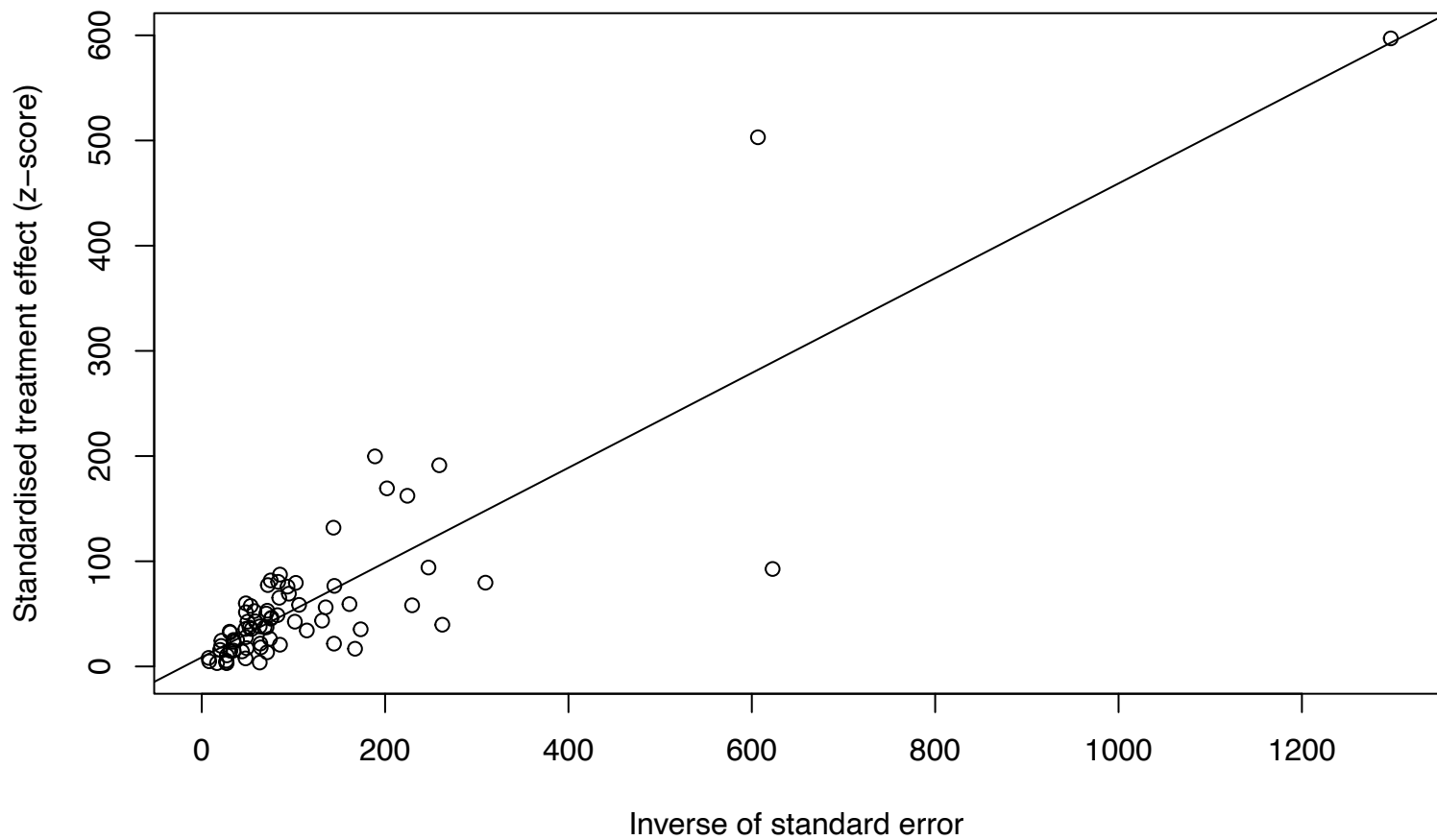

Figure S3 Egger's test for the publication bias

Supplement: Supplementary file 3 [file DataSheet_3.pdf]

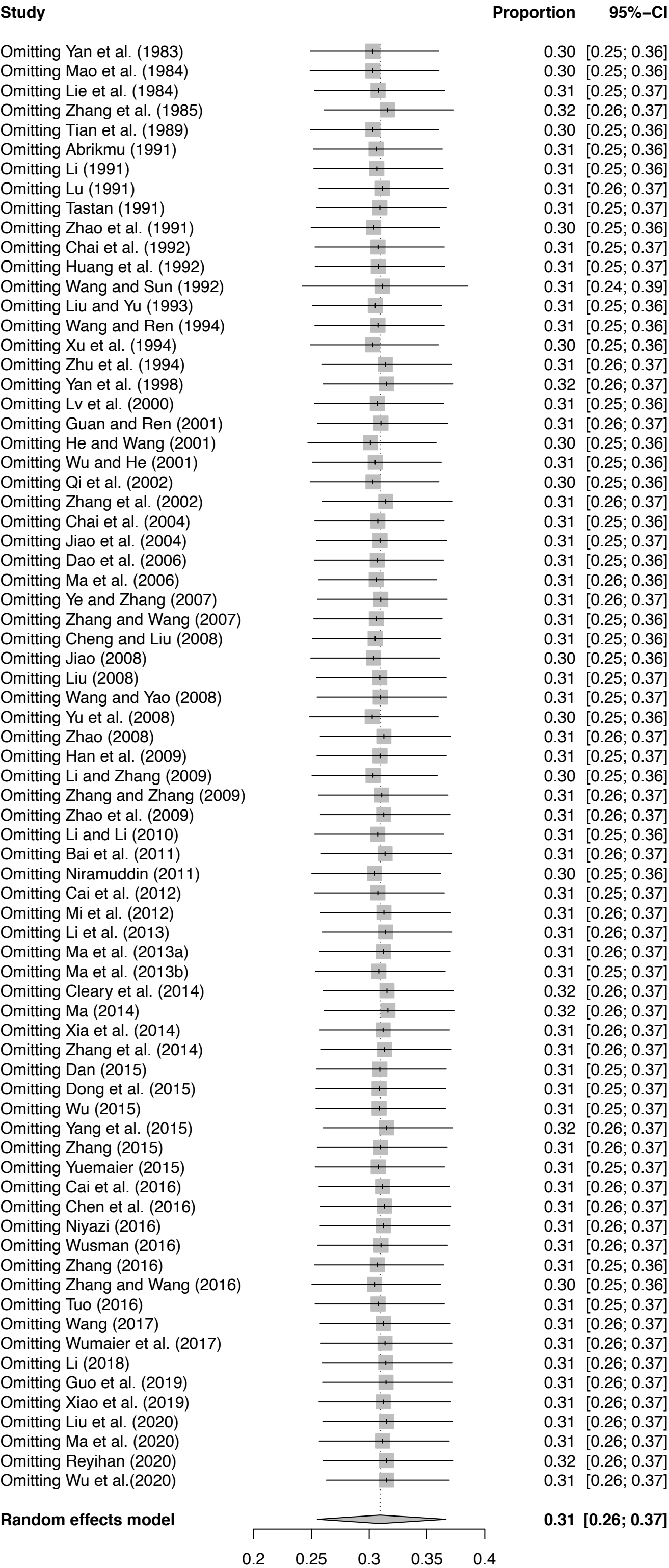

Figure S4 Sensitivity analysis

Supplement: Supplementary file 4 [file DataSheet_4.pdf]
