# Supplementary material for: Meta-Analysis of the Prevalence of Echinococcus in Sheep in China From 1983 to 2020
Source: Front Cell Infect Microbiol. 2021 Jul 26;11:711332. doi: 10.3389/fcimb.2021.711332 (PMC8350519; doi:10.3389/fcimb.2021.711332)
Supplement: Supplementary file 5 [file DataSheet_5.doc]

**TABLE S1** **|** The code in R for meta-analysis.

| Logarithmic conversion (PNL) | rate<-transform [m1, log=log(event/n)];  shapiro.test(rate$log) |
| --- | --- |
| Logit transformation (PLOGIT) | rate<-transform{m1, logit=log[(event/n)/(1-event/n)]};  shapiro.test(rate$logit) |
| Arcsine transformation (PAS) | rate<-transform{m1, arcsin.size=asin[sqrt(event/(n+1))]};  shapiro.test(rate$arcsin) |
| Double-arcsine transformation (PFT) | rate<-transform{m1,darcsin=0.5*[asin(sqrt(event/(n+1)))+asin((sqrt(event+1)/(n+1)))]};  shapiro.test(rate$darcsin) |
| No transformation (PRAW) | rate<-transform[m1, r= event/n];  shapiro.test(rate$r) |
| Forest plots | forest [meta1, xlim=c(-0.2, 1)] |
| Funnel chart | funnel (meta1) |
| Egger's test | metabias (meta1, method="linreg") |
| The sensitivity analysis | metainf (meta1, pooled = "random") forest (metainf (meta1, pooled = "random"), xlim=c(0.2, 0.4)) |
| Subgroup analysis | meta1<-metaprop(event, n, study, data=rate, sm="PFT", incr=0.5, allincr=TRUE, addincr=FALSE, title="", byvar= subgroup title, print.byvar=TRUE) |
| Meta-regression analysis | metareg (meta1, ~covariate title) |

**TABLE S2 | Included studies and quality scores**.

| **Reference ID** | **No.**  **tested** | **No. positive** | **Prevalence** | **Random sampling**  **or not** | **Clear detection method or not** | **Sampling method in detail or not** | **Sampling time clearly or not** | **4 or more risk factors or not** | **Score** | **Study**  **Quality** |
| --- | --- | --- | --- | --- | --- | --- | --- | --- | --- | --- |
| Yan et al. (1983) | 711 | 552 | 77.6% | N | N | N | N | Y | 1 | Low |
| Lie et al. (1984) | 12,577 | 5,510 | 43.8% | N | Y | Y | Y | N | 3 | Medium |
| Mao et al. (1984) | 231 | 181 | 78.4% | N | Y | N | Y | Y | 3 | Medium |
| Zhang et al. (1985) | 6,989 | 70 | 1.0% | Y | Y | Y | Y | N | 4 | High |
| Tian et al. (1989) | 1,300 | 1,003 | 77.2% | N | Y | N | N | Y | 2 | Medium |
| Abrikmu (1991) | 623 | 353 | 56.7% | N | Y | N | N | Y | 2 | Medium |
| Li (1991) | 2,189 | 1,150 | 52.5% | N | Y | N | N | Y | 2 | Medium |
| Tastan (1991) | 1,000 | 320 | 32.0% | Y | Y | Y | Y | Y | 5 | High |
| Zhao et al. (1991) | 1,820 | 1,327 | 72.9% | N | Y | N | Y | Y | 3 | Medium |
| Chai et al. (1992) | 2,258 | 999 | 44.2% | N | Y | Y | Y | Y | 4 | High |
| Huang et al. (1992) | 680 | 288 | 42.4% | N | Y | N | Y | Y | 3 | Medium |
| Wang and Sun (1992) | 420,507 | 83,004 | 19.7% | N | N | N | Y | Y | 2 | Medium |
| Liu and Yu (1993) | 112 | 71 | 63.4 | Y | Y | N | N | N | 2 | Medium |
| Wang and Ren (1994) | 854 | 382 | 44.7% | Y | Y | Y | N | N | 3 | Medium |
| Xu et al. (1994) | 1,413 | 1,108 | 78.4% | Y | Y | Y | Y | N | 4 | High |
| Zhu et al. (1994) | 1,820 | 104 | 5.7% | Y | N | N | Y | N | 2 | Medium |
| Yan et al. (1998) | 178 | 3 | 1.7% | N | Y | Y | N | N | 2 | Medium |
| Lv et al. (2000) | 2,632 | 1,287 | 48.9% | N | N | N | Y | Y | 2 | Medium |
| Guan and Ren (2001) | 5,231 | 1,334 | 25.5% | N | Y | N | N | Y | 2 | Medium |
| He and Wang (2001) | 578 | 520 | 90.0% | N | Y | N | Y | Y | 3 | Medium |
| Wu and He (2001) | 825 | 519 | 62.9% | N | Y | Y | Y | Y | 4 | High |
| Qi et al. (2002) | 580 | 446 | 76.9% | N | Y | N | Y | Y | 3 | Medium |
| Zhang et al. (2002) | 69 | 2 | 2.9% | N | Y | Y | N | Y | 3 | Medium |
| Chai et al. (2004) | 1,278 | 582 | 45.5% | N | Y | N | Y | Y | 3 | Medium |
| Jiao et al. (2004) | 1,705 | 528 | 31.0% | N | Y | N | N | Y | 2 | Medium |
| Dao et al. (2006) | 100 | 50 | 50.0% | N | Y | N | N | Y | 2 | Medium |
| Ma et al. (2006) | 92,015 | 50,037 | 54.4% | N | Y | N | Y | Y | 3 | Medium |
| Ye and Zhang (2007) | 1,175 | 313 | 26.6% | Y | Y | N | Y | Y | 4 | High |
| Zhang and Wang (2007) | 10,204 | 5,638 | 55.3% | Y | Y | Y | Y | Y | 5 | High |
| Cheng and Liu (2008) | 5,160 | 3,257 | 63.1% | N | Y | N | Y | Y | 3 | Medium |
| Jiao (2008) | 240 | 179 | 74.6% | N | N | N | N | Y | 1 | Low |
| Liu (2008) | 1,450 | 472 | 32.6% | Y | Y | N | Y | Y | 4 | High |
| Wang and Yao (2008) | 581 | 173 | 29.8% | Y | Y | N | Y | Y | 4 | High |
| Yu et al. (2008) | 115 | 95 | 82.6% | N | N | N | Y | Y | 2 | Medium |
| Zhao (2008) | 4,309 | 457 | 10.6% | N | Y | Y | Y | Y | 4 | High |
| Han et al. (2009) | 16 | 5 | 31.3% | N | Y | N | Y | Y | 3 | Medium |
| Li and Zhang (2009) | 8,927 | 6,769 | 75.8% | N | Y | N | Y | Y | 3 | Medium |
| Zhang and Zhang (2009) | 243 | 51 | 21.0% | N | Y | N | Y | Y | 3 | Medium |
| Zhao et al. (2009) | 1,021 | 113 | 11.1% | N | Y | N | Y | Y | 3 | Medium |
| Li and Li (2010) | 568 | 262 | 46.1% | N | Y | N | Y | N | 2 | Medium |
| Bai et al. (2011) | 13,160 | 826 | 6.3% | N | Y | N | Y | Y | 3 | Medium |
| Niramuddin (2011) | 1,738 | 1,174 | 67.5% | N | Y | Y | Y | Y | 4 | High |
| Cai et al. (2012) | 16,785 | 7,600 | 45.3% | N | Y | N | Y | Y | 3 | Medium |
| Mi et al. (2012) | 482 | 49 | 10.2% | Y | Y | N | Y | Y | 4 | High |
| Li et al. (2013) | 180 | 7 | 3.9% | N | Y | Y | Y | Y | 4 | High |
| Ma et al. (2013a) | 6,490 | 838 | 12.9% | N | Y | N | Y | Y | 3 | Medium |
| Ma et al. (2013b) | 300 | 117 | 39.0% | N | Y | N | Y | Y | 3 | Medium |
| Cleary et al. (2014) | 184 | 2 | 1.1% | Y | Y | Y | N | Y | 4 | High |
| Ma (2014) | 1,000 | 3 | 0.3% | N | Y | Y | Y | Y | 4 | High |
| Xia et al. (2014) | 188 | 26 | 13.8% | Y | Y | N | N | Y | 3 | Medium |
| Zhang et al. (2014) | 1,035 | 79 | 7.6% | N | Y | N | Y | Y | 3 | Medium |
| Dan (2015) | 1,418 | 456 | 32.2% | N | N | N | Y | N | 1 | Low |
| Dong et al. (2015) | 371 | 137 | 36.9% | N | Y | N | Y | Y | 3 | Medium |
| Wu (2015) | 742 | 274 | 36.9% | N | Y | N | Y | Y | 3 | Medium |
| Lu (2015) | 302 | 53 | 17.5% | N | N | N | Y | Y | 2 | Medium |
| Yang et al. (2015) | 17,215 | 390 | 2.3% | N | Y | N | Y | Y | 3 | Medium |
| Yuemaier (2015) | 1,237 | 531 | 42.9% | Y | Y | Y | Y | N | 4 | High |
| Zhang (2015) | 2,819 | 773 | 27.4% | N | Y | N | Y | Y | 3 | Medium |
| Cai et al. (2016) | 4,568 | 745 | 16.3% | Y | Y | N | N | Y | 3 | Medium |
| Chen et al. (2016) | 3,283 | 283 | 8.6% | N | Y | N | Y | Y | 3 | Medium |
| Niyazi (2016) | 600 | 72 | 12.0% | N | Y | Y | Y | Y | 4 | High |
| Tuo (2016) | 300 | 132 | 44.0% | Y | Y | Y | Y | Y | 5 | High |
| Wusman (2016) | 1,250 | 315 | 25.2% | N | Y | N | N | Y | 2 | Medium |
| Zhang (2016) | 1,787 | 869 | 48.6% | Y | Y | Y | Y | Y | 5 | High |
| Zhang and Wang (2016) | 14 | 11 | 78.6% | N | Y | Y | Y | Y | 4 | High |
| Wang (2017) | 1,383 | 165 | 11.9% | N | Y | Y | Y | Y | 4 | High |
| Wumaier et al. (2017) | 23,943 | 1,550 | 6.5% | N | Y | N | Y | Y | 3 | Medium |
| Li (2018) | 7,513 | 307 | 4.1% | Y | Y | Y | Y | Y | 5 | High |
| Guo et al. (2019) | 1,270 | 44 | 3.5% | N | Y | N | Y | Y | 3 | Medium |
| Xiao et al. (2019) | 15,287 | 2,106 | 13.8% | Y | Y | Y | Y | Y | 5 | High |
| Liu et al. (2020) | 571 | 14 | 2.5% | N | Y | Y | N | Y | 3 | Medium |
| Ma et al. (2020) | 2,579 | 426 | 16.5% | Y | Y | N | N | Y | 3 | Medium |
| Reyihan (2020) | 5,202 | 116 | 2.2% | N | Y | N | Y | Y | 3 | Medium |
| Wu et al. (2020) | 96,926 | 2,130 | 2.2% | N | Y | N | Y | Y | 3 | Medium |

**References**

Abrikmu, Y. (2000). Investigation of hydatid disease infection in sheep slaughtered in Zhonghuan Road Slaughterhouse in Urumqi. *Rural. Sci. Technol.* 7, 23. (In Chinese)

Bai, T.J., Yang, W.D., Bai, T.X., Ma, Y.S. (2011). Investigation and control measures of hydatid infection in sheep in Tianzhu County. *Anim. Husb. Vet. Med.* 61, 73-5. (In Chinese)

Cai, H.X., Wang, H., Han, X.M., Ma, X., Zhang, J.X., Liu, Y.F., et al. (2016). Investigation on epidemic status of echinococcosis in Hainan Tibetan Autonomous Prefecture of Qinghai Province. *J. Pathog. Biol.* 11, 1022-5. (In Chinese)

Cai, H.X., Wang, H., Han, X.M., Ma, X., Liu, Y.F., Liu, P.Y., et al. (2012). Study on the infection status and significance of different hosts of *Echinococcus* in Qinghai Plateau. *Chin. J. Endemiol.* 31, 296-300. (In Chinese)

Chai, J.J., Jiao, W., Yi, S.L.Y., Chang, Q., Meng, H.B.T., Fu, C., et al. (2004). Epidemic status of cystic echinococcosis in Northern Xinjiang. *J. Trop. Dis. Parasitol.* 2, 139-43. (In Chinese)

Chai, J.J., Jiao, W., Zhang, W.L., Yi, S.L.Y., Qu, Q., Li, X.L., et al. (1992). Comparative observation on infection characteristics of *Echinococcus* *granulosus* in cattle and sheep in different areas of Xinjiang. *Bull. Dis. Control Prev.* S1, 1-7. (In Chinese)

Chen, X.Y., Setiwaldi, Y., Osman, Y. (2016). Epidemiological studies on echinococcosis in Kizilsu Kirgiz Autonomous Prefecture of Xinjiang. *Chin. J. Parasitol. Parasitic Dis.* 34, 409-13. (In Chinese)

Cheng, H.P., Liu, X.R. (2008). Investigation on infection of two types of hydatid disease in plateau animals in Qingnan area. *J. High Altitude Med.* 18(002), 56-8. (In Chinese)

Cleary, E., Barnes, T.S., Xu, Y., Zhao, H., Clements, A.C., Gray, D.J., et al. (2014). Impact of "Grain to Green" programme on echinococcosis infection in Ningxia Hui Autonomous Region of China. *Vet. Parasitol.* 205, 523-31. doi: 10.1016/j.vetpar.2014.08.023

Dan, Z.C. (2015). Analysis on control effect of echinococcosis among livestock in Guinan County of Qinghai Province. *Anim Husb. Feed Sci.* 36, 125-6. (In Chinese)

Dao, J.C., Ge, D., Wan, D.C. (2006). Epidemiological investigation of hydatid disease in Maqu County, Gannan Prefecture. *Chin. Anim. Health* 9, 34-6. (In Chinese)

Dong, J., Yang, L.F., Zhang, W.B., Li, H.T., Jiang, T., Qi, X.W., et al. (2015). Prevalence rate of ovine hepatic cystic echinococcosis in Quaker Wusu area of Bayinbuluke of Xinjiang, 2014. *Chin. J. Epidemiol.* 36, 136-8. (In Chinese)

Guan, G., Ren, G.J. (2001). Investigation on the infection of hydatidosis in cattle and sheep slaughtered in Golmud. *Chin.* *Anim. Health Inspection* 09, 33-4. (In Chinese)

Guo, B.P., Zhang, Z.Z., Zheng, X.T., Guo, Y.Z., Guo, G., Zhao, L., et al. (2019). Prevalence and molecular characterization of *Echinococcus* *granulosus* sensu stricto in Northern Xinjiang, China. *Korean J. Parasitol.* 57, 153-9. doi: 10.3347/kjp.2019.57.2.153

Han, X.M., Wang, H., Cai, H.X., Ma, X., Liu, Y.F., Wei, B.H., et al. (2009). Epidemiological survey on echinococcosis in Darlag County of Qinghai Province. Chin. *J. Parasitol Parasitic Dis.* 27, 22-6. (In Chinese)

He, D.L., Wang, H. (2001). Epidemiological evaluation report of hydatid disease in Zeku County, Qinghai Province. *Bull. Dis. Control Prev.* 36-8. (In Chinese)

Huang, C.G., Chao, J.T. (1992). About Heimahe and five other villages' cattle sheep *Echinococcus* infections and hazard investigation. *Qinghai Husbandary*. 3, 25-6. (In Chinese)

Jiao, F.R. (2008). Investigation on epidemic status of echinococcosis in Xilinguole League. *Bull. Dis. Control Prev.* 2,46+48. (In Chinese)

Jiao, W., Fu, C., Qu, Q., Nu, E.B.K., Xu, S.D., Sun, L.F., et al. (2004). Epidemiological evaluation on the efficacy of the slowly released bar of parziquantel for dog use in the prevention of cystic echinococcosis in human and sheep. *Chin. J. Zoonoses.* 20, 557-60. (In Chinese)

Li, H.T., Song, T., Duan, X.Y., Qi, X.W., Feng, X.H., Wang, Y.H., et al. (2013). Liver echinococcosis screening report of population and flock in Xinjiang and Buxaer Mongolian Autonomous County. *Chin. J. Epidemiol.* 34, 1176-8. (In Chinese)

Li, J.F. (2018). Epidemiological investigation of sheep echinococcosis in Yumen City. *Gansu Agr. Univ.* 50. (In Chinese)

Li, L.Z., Zhang, L.C. (2009). Investigation and control of echinococcosis in Tibetan Sheep. *Chin. Anim. Husb. Vet.* 36, 149-50. (In Chinese)

Li, Q.Q. (2009). Investigation on *Echinococcus* infection in cattle and sheep in Ulan County. *Chin. Qinghai J. Anim. Vet. Sci.* 39, 3. (In Chinese)

Li, Y.F., Li, D.S. (2010). Investigation on epidemic situation of echinococcosis in Haiyan area. *Anim. Quarantine in Chin.* 27, 38. (In Chinese)

Lie, T.F., Li, Q.C., Zhou, J.H., Gongbao, D.J. (1984). Investigation Report on *Echinococcus* Infection in Cattle and Sheep in Gande Area. *Qinghai J Anim Husb Vet Med*, 19-21. (In Chinese)

Liu, K.Y., Guan, C., Liu, Y.H., Fang, S.F., Cui, P. (2020). Investigation on sheep hydatid infection in Zhangjiakou City. *Chin. Herbiv. Sci.* 40, 89-90. (In Chinese)

Liu. X.T, Yu. D.J. (1994). Preliminary investigation of animal echinococcosis infection in Naqu area. *Chin J Parasito Parasiti Dis*, 77. (In Chinese)

Liu, Y.M. (2008). Investigation of sheep hydatid infection in Chaka District. *Chin.* *Qinghai J. Anim. Vet. Sci.* 39, 34. (In Chinese)

Lu, Y. (2015). Investigation and control of sheep hydatid disease in Gangcha County. *Contemp. Anim. Husb..* 33, 61-2. (In Chinese)

Lv, C.F., Li, X.Y., Li, S.Y., Li, X. (2000). Investigation of hydatid disease in sheep in Republic area. *Chin. Qinghai J. Anim. Vet. Sci.* 28, 48. (In Chinese)

Ma, B., Su, X.Y., Batai, M., Joe, G. (2020). Epidemiological investigation of sheep hydatid disease in some areas of Bazhou. *Mod. J. Anim. Husb. Vet. Med.* 49, 45-8. (In Chinese)

Ma, L.K., Lin, H.L., Nu, S.L.T., Zuli, H.M.E., Yan, H., Ba, T.L., et al. (2013). Comprehensive prevention and control measures for livestock hydatid disease in Burqin County. *Zhongguo Xumu Shouyi Wenzhai* 29, 85-6. (In Chinese)

Ma, L.P., Shen, X., He, G.M. (2013). Investigation and analysis of echinococcosis in sheep in slaughterhouse in Wusu City, Xinjiang. *Beijing Agr.* 32, 181-2. (In Chinese)

Ma, S.M., Wang, H., Li, W.M. (2006). Analysis of hydatid disease data in Qingnan area from 1997 to 2001. *J. Trop. Med.* 28, 55-7. (In Chinese)

Ma, Y.L. (2014). Screening and analysis of hydatid disease in Hualong County of Qinghai Province in 2012. *Chin. J. Pest Control* 5, 577. (In Chinese)

Mao, G.H., Weng, J.Y., Cao, Y.Q. (1984). Investigation on echinococcosis of yak and Tibetan sheep in Shiqu County, Sichuan Province. *Chin. Vet. Sci.* 33-4. (In Chinese)

Mi, X.L., Li, D.M., Feng, S.Q., Zhou, Y.Q., Gao, J.M. (2012). Investigation of echinococcosis in cattle and sheep in Menyuan county. *Chin. Qinghai* *J. Anim. Vet. Sci.* 42, 31-2. (In Chinese)

Niramuddin, A. (2011). Comparative observation on the infection characteristics of echinococcosis in sheep and cattle in northern and southern Xinjiang. *Chin. J. Vet. Med.* 47, 81-2. (In Chinese)

Niyazi, A. (2016). Investigation on echinococcosis infection of livestock in Kuqa County, Xinjiang. *Anim. Health.* 10, 115-115. (In Chinese)

Qi, Y.Z., Wen, Z.Q., Song, T., Wang, Y.S. (2002). Epidemiological investigation of hydatidosis in Tianzhu Tibetan Autonomous County, Gansu Province. *Endemic. Dis. Bull.* 18, 51-3. (In Chinese)

Reyihan, Y.S.F. (2020). Molecular epidemiological investigation of cattle and sheep echinococcosis in parts of Xinjiang. *Tarim. Univ.* (In Chinese)

Tastan, A. (2011). Investigation and research on the infection situation of cystic ec hinococcosis in sheep in the surrounding area of Yining. *Xinjiang Agr. Univ.* (In Chinese)

Tian, G.F., Li, Z.H., Liu, J.F., Jia, W.Z., Ye, P.Z., Ming, R.J., et al. (1989). Epidemiological investigation of hydatid disease in Huangcheng sheep farm in Gansu Province. *Chin. Vet. Sci.* 13-6. (In Chinese)

Tuo. J.Z. (2016). Investigation and control of hydatid disease in sheep. *Chin Anim Husb Vet Digest* 32, 121. (In Chinese)

Wang, F.Y., Sun, H.S. (1992). Investigation and analysis of sheep echinococcosis in Hami Area. *Meat Hyg.* 9, 12-3. (In Chinese)

Wang, H.G., Yao, H.R. (2008). Investigation on *Echinococcus* infection of sheep in Zhaba town of Hualong County. Chin. *Qinghai J. Anim. Vet. Sci.* 38, 44. (In Chinese)

Wang, L.M. (2017). Epidemiological investigation of *Echinococcus* infection and it’s influences on sheep in different terrains in Akesu Prefecture. *Tarim. Univ.* (In Chinese)

Wang, J.G., Ren, D.Y. (1994). Investigation on Hydatid Infection of Cattle and Sheep in Xiahe County, Gansu Province. *Chin Vet Sci Technol*, 17-18. (In Chinese)

Wu, A.M. (2015). Survey on the prevalence of hepatic cystic hydatid disease in sheep in Emin County, Xinjiang in 2014. *Psychol. Doctor* 21, 240-1. (In Chinese)

Wu, X.H., He, D.L. (2001). An epidemiological investigation on hydatid disease in Gonghe County, Qinghai Province. *Bull. Dis. Control Prev. (China).* 29-31. doi: 10.13215/j.cnki.jbyfkztb.2001.01.012 (In Chinese)

Wu, X.L., Duan, H.J., Qi, R.T., Yan, F., Fu, Y.R., Ma, T.B. (2020). Evaluation of the effect of the integrated echinococcosis control program in Ningxia Hui Autonomous Region from 2011 to 2018. *Zhongguo Xue Xi Chong Bing Fang Zhi Za Zhi* 32, 598-604. (In Chinese)

Wumaier, M., Usman, I., Simayi, A., Hou, Y.Y, Xiao, N. (2017). Investigation and analysis of animal *Echinococcus* infection in Xinjiang Uygur Autonomous Region. *Chin. J. Parasitol. Parasitic Dis.* 35, 145-9. (In Chinese)

Wusman, A. (2016). Detection and epidemiological analysis of aspidistra spinosa in livestock slaughterhouse of Kuqa County. *Chin. Anim. Husb. Vet. Abstr.* 111. (In Chinese)

Xia, C.Y., Liu, J.Z., Tsering, D., Yuan, Z.J., Gesang, D., Ban, D., et al. (2014). Investigation of three finds of tapeworm larvae infections in Tibetan domestic animals. *Chin. Vet. Sci.* 44, 1205-9. (In Chinese)

Xiao, G.L., Zhong, Q., Xie, W.H., Wang, X.H. (2019). Epidemiological survey of sheep hydatidosis in Kashi area of Xinjiang from 2014 to 2017. *Chin. Anim. Health Inspection* 36, 1-5. (In Chinese)

Xu, X.P., Li, X.J., Zhu, H.C., Tao, Y., Li, Y.X. (1994). Investigation on current situation of hydatid disease in Shihezi area. *Shihezi Technol*, 64-65. (In Chinese)

Yan, H.C., Zhou, Z.S., Liu, X.G., Du, H. (1998). Investigation on parasite infection in sheep lungs. *Meat Hyg.* 21, 6. (In Chinese)

Yan, Q.L., Wei, M.L., Fan, C.B., Cai, D., Shi, Y. (1983). Investigation report on echinococcosis infection of cattle and sheep in Huangnan Prefecture of Qinghai Province. *Chin. Qinghai J. Anim. Vet. Sci.* 4, 34-6. (In Chinese)

Yang, S.J., Wu, W.P., Tian, T., Zhao, J.S., Wang, Q.Y., Zheng, F. (2015). Prevalence of cystic echinococcosis in slaughtered sheep as an indicator to assess control progress in Emin County, Xinjiang, *Chin. Korean. J. Parasitol.* 53, 355-9. dio: 10.3347/kjp.2015.53.3.355

Ye, W.X., Zhang, X.G. (2007). Investigation of hydatid infection in sheep in Minhe County. *Chin. Qinghai J. Anim. Vet. Sci.* 27. (In Chinese)

Yu, S.H., Wang, H., Wu, X.H., Ma, X., Liu, Y.F., Zhao, Y.M., et al. (2008). Cystic and alveolar echinococcosis: an epidemiological survey in a Tibetan population in Southeast Qinghai, China. *Jpn. J. Infect. Dis.* 61, 242-6.

Yuemaier, T. (2015). Investigation on the infection status of domestic animal hydatid disease in Wushi County, Xinjiang. *Vet Guide* 000, 153-153. (In Chinese)

Zhang, F., Xu, W.P., Wang, H.P., Zhang, Q.H., Yan, R.L. (1985). Preliminary investigation on the epidemiology of polycephalytaeniasis in Ningxia. *Ningxia Agri Sci Technol*, 35-36. (In Chinese)

Zhang, H.C., Zhang, Y.Q. (2009). Investigation of hydatid infection in dogs and sheep in Zhugu Township, Menyuan County. *Anim. Husb. Vet. Med.* 41, 109. (In Chinese)

Zhang, H.W., Ding, Y.L., Qiao, T.S. (2002). A survey on the parasite system of sheep in Shuangcheng City, Heilongjiang Province. *Chin. Vet. Sci.* 32, 15-6. (In Chinese)

Zhang, J.X., Wang, H. (2007). Epidemiological survey on *Echinococcus* infection in animals in Qinghai Province. *Chin. J. Parasitol. Parasiti. Dis.* 24, 350-2. (In Chinese)

Zhang, K.F., Wu, T.J., Zhao, Z.Y. (2014). Analysis of surveillance results of hydatid disease in Minle County from 2009 to 2010. *The Fifth Cross-Strait (Qinghai) Featured Agr. Industrial. Forum* 258-60. (In Chinese)

Zhang, L.Y., Wang, X.F. (2016). Investigation and research on endoparasites of cattle and sheep in Tuergate Port Area. *Xinjiang Anim. Husb.* 7, 28-30. (In Chinese)

Zhang, R. (2016). Detection of individual prevalence of sheep hydatid disease in Wusu City, Xinjiang. *Xinjiang. Agr. Univ.* (In Chinese)

Zhang, X. (2015). Demonstration and application of hydatid detection and comprehensive prevention and control measures for hydatid disease. *Xinjiang Agr. Univ.* (In Chinese)

Zhao, H.J., An, H.H., Wu, D.X., Ro, Z.G.L., Ka, H., Shen, Y., et al. (1991). Investigation of sheep hydatid disease in Yiwu County. *Xinjiang Husb..* 7, 15-7. (In Chinese)

Zhao, Y.M. (2008). Epidemiological study on hydatid disease in the eastern part of the Qinghai-Tibet Plateau (Gannan Tibetan Autonomous Prefecture). *Gansu. Agr. Univ.* (In Chinese)

Zhao, Y.M., Tong, S.X., Jing, T., Zhong, S.G., Cai, X.P., Jing, Z.Z., et al. (2009). Investigation on echinococcosis in animals in Gannan Tibetan Autonomous Prefecture. *Chin. J. Parasitol. Parasitic. Dis.* 27, 27-30. (In Chinese)

Zhu, G.L., Guo, X.H., Liu, Z.Q., Wu, Q.W., Li, L.F. (1994). Investigation on Hydatid Disease of Sheep in Southern Xinjiang Reclamation Area. *Heilongjiang Anim Husb Vet*, 26-27. (In Chinese)

**TABLE S3** **|** Egger’s for publication bias.

| **slope** | **bias** | **se. bias** | **t** | **df** | ***P*-value** |
| --- | --- | --- | --- | --- | --- |
| 0.451 | 8.683 | 6.578 | 1.320 | 72 | 0.0311 |

**TABLE S4** **|** Normal distribution test for the normal rate and the different conversion of the normal rate.

| **Conversion form** | ***W*** | ***P*** |
| --- | --- | --- |
| **PRAW** | 0.924 | 0.000 |
| **PLN** | 0.884 | 5.969e-06 |
| **PLOGIT** | 0.971 | 0.087 |
| **PAS** | 0.962 | 0.025 |
| **PFT** | 0.963 | 0.031 |

“PRAW”: original rate; “PLN”: logarithmic conversion; “PLOGIT”: logit transformation; “PAS”: arcsine transformation; “PFT”: double-arcsine transformation

**TABLE S5** **|** Sub-group analysis of the prevalence of *Echinococcus* according to geographic location and climate variables.

|  |  | **No. studies** | **No. examined** | **No. positive** | **% (95% CI*)** | **Heterogeneity**  **Univariate meta-regression** | | | **Univariate meta-regression** | |
| --- | --- | --- | --- | --- | --- | --- | --- | --- | --- | --- |
|  |  |  |  |  |  | ***χ*^2^** | ***P*-value** | ***I^2^* (%)** | ***P*-value** | **Coefficient (95% CI)** |
| **Latitude** |  |  |  |  |  |  |  |  | 0.0017 | 0.219 (0.083 to 0.356) |
|  | 30–35° | 18 | 58,468 | 20,223 | 46.9% (29.5–64.6) | 28,588.7 | 0.00 | 99.9 |  |  |
|  | 36–40° | 28 | 180,358 | 68,815 | 24.5% (15.3–35.0) | 49,339.1 | 0.00 | 99.9 |  |  |
|  | 41*–*45° | 17 | 461,427 | 91,354 | 31.8% (22.9–41.3) | 13,198.3 | 0.00 | 99.9 |  |  |
|  | 46*–*50° | 11 | 15,994 | 3,731 | 21.5% (12.9–31.6) | 1,844.4 | 0.00 | 99.5 |  |  |
| **Longitude** |  |  |  |  |  |  |  |  | 0.0504 | 0.148 (-0.000 to 0.296) |
|  | 75*–*80° | 10 | 26,745 | 4,229 | 22.7% (13.5–33.4) | 2,429.0 | 0.00 | 99.6 |  |  |
|  | 81–90° | 21 | 69,222 | 9,859 | 27.3% (18.2–37.5) | 14,492.0 | 0.00 | 99.9 |  |  |
|  | 91–100° | 22 | 480,554 | 108,639 | 42.7% (32.3–53.4) | 29,188.2 | 0.00 | 99.9 |  |  |
|  | 101*–*126° | 23 | 139,729 | 61,396 | 31.9% (19.8–45.4) | 34,549.7 | 0.00 | 99.9 |  |  |
| **Rainfall** (mm) |  |  |  |  |  |  |  |  | 0.0004 | 0.197 (0.089 to 0.306) |
|  | 0–100 | 7 | 430,328 | 85,010 | 17.8% (8.6–29.4) | 2,640.6 | 0.00 | 99.8 |  |  |
|  | 101*–*200 | 15 | 59,688 | 9,147 | 24.9% (14.2–37.3) | 12,969.9 | 0.00 | 99.9 |  |  |
|  | 201*–*400 | 22 | 56,618 | 9,478 | 27.7% (18.3–38.3) | 13,304.6 | 0.00 | 99.8 |  |  |
|  | 401*–*1,000 | 16 | 143,001 | 74,458 | 43.5% (34.9–52.4) | 10,129.6 | 0.00 | 99.9 |  |  |
| **Temperature** (℃) |  |  |  |  |  |  |  |  | 0.0015 | 0.321 (0.122 to 0.520) |
|  | -5–0 | 7 | 130,003 | 64,991 | 58.7% (23.9–89.1) | 16,039.3 | 0.00 | 100.0 |  |  |
|  | 0–5 | 24 | 129,760 | 64,940 | 31.7% (24.3–39.6) | 10,441.7 | 0.00 | 99.8 |  |  |
|  | 6–10 | 21 | 507,136 | 99,033 | 27.1% (19.8–35.1) | 24,494.9 | 0.00 | 99.9 |  |  |
|  | 11–17 | 8 | 22,654 | 3,466 | 15.8% (8.3–25.1) | 1,758.3 | 0.00 | 99.6 |  |  |
| **Humidity** (%) |  |  |  |  |  |  |  |  | 0.285 | 0.185 (0.028 to 0.341) |
| (%) | 25–46 | 11 | 451,065 | 89,970 | 31.7% (22.6–41.5) | 6,646.0 | 0.00 | 99.8 |  |  |
|  | 47–55 | 19 | 48,358 | 7,530 | 25.9% (15.7–37.6) | 12,241.0 | 0.00 | 99.9 |  |  |
|  | 56– 60 | 18 | 152,366 | 69,835 | 33.1% (20.5–47.0) | 34,535.2 | 0.00 | 100.0 |  |  |
|  | 61– 68 | 12 | 36,059 | 9,889 | 38.5% (22.3–56.0) | 10,950.2 | 0.00 | 99.9 |  |  |
| **Average annual minimum** |  |  |  |  |  |  |  |  | < 0.0001 | 0.438 (0.273 to 0.604) |
| **temperature** (℃) | < -5 | 9 | 27,621 | 16,414 | 68.8% (55.9–80.3) | 3,053.8 | 0.01 | 99.7 |  |  |
|  | -4–0 | 23 | 131,698 | 61,373 | 32.1% (21.7–43.6) | 21,671.7 | 0.00 | 99.9 |  |  |
|  | 0–5 | 21 | 502,414 | 95,999 | 22.7% (16.3–29.9) | 21,179.4 | 0.00 | 99.9 |  |  |
|  | 6–12 | 6 | 20,671 | 3,229 | 17.1% (7.6–29.5) | 1,757.5 | 0.00 | 99.7 |  |  |
| **Average annual maximum** |  |  |  |  |  |  |  |  | 0.0002 | 0.289 (0.139 to 0.440) |
| **temperature** (℃) | 0–10 | 14 | 49,436 | 19,655 | 53.0% (35.0–70.6) | 19,421.6 | 0.00 | 99.9 |  |  |
|  | 11–13 | 17 | 18,543 | 7,646 | 26.0% (16.2–37.2) | 4,227.6 | 0.00 | 99.6 |  |  |
|  | 14–16 | 20 | 173,916 | 63,138 | 26.3% (14.3–40.5) | 55,271.6 | 0.00 | 100.0 |  |  |
|  | > 17 | 10 | 447,740 | 87,654 | 21.5% (14.2–29.8) | 4,928.9 | 0.00 | 99.8 |  |  |
| **Altitude** (0.1 m) |  |  |  |  |  |  |  |  | 0.0004 | 0.244 (0.110 to 0.378) |
|  | 300–10,000 | 24 | 489,298 | 93,568 | 25.5% (19.0–32.5) | 18,730.6 | 0.00 | 99.9 |  |  |
|  | 10,001–15,000 | 17 | 41,311 | 6,591 | 24.0% (15.4–33.8) | 6,955.4 | 0.00 | 99.8 |  |  |
|  | 15,001– 30,000 | 17 | 128,725 | 62,138 | 33.3% (21.3–46.5) | 18,840.9 | 0.00 | 99.9 |  |  |
|  | 30,001– 100,000 | 18 | 56,913 | 21,826 | 50.7% (35.6–65.8) | 20,815.2 | 0.00 | 99.9 |  |  |

CI*: Confidence interval
